# Frequency and Clinical Significance of Clonal and Subclonal Driver Mutations in High-Risk Neuroblastoma at Diagnosis: A Children's Oncology Group Study

**DOI:** 10.1200/JCO-24-02407

**Published:** 2025-03-04

**Authors:** Esther R. Berko, Arlene Naranjo, Alexander A. Daniels, Samantha N. McNulty, Kateryna Krytska, Todd Druley, Kirstin Zelley, Balakrishna Koneru, Lulu Chen, Grace Polkosnik, Meredith S. Irwin, Rochelle Bagatell, John M. Maris, C. Patrick Reynolds, Steven G. DuBois, Julie R. Park, Yael P. Mossé

**Affiliations:** ^1^ Division of Oncology and Center for Childhood Cancer Research, Children's Hospital of Philadelphia, Philadelphia, PA; ^2^ Division of Hematology-Oncology, Schneider Children's Medical Center of Israel, Petach Tikvah, Israel; ^3^ Faculty of Medicine, Tel Aviv University, Tel Aviv, Israel; ^4^ Department of Biostatistics, Children's Oncology Group Statistics and Data Center, University of Florida, Gainesville, FL; ^5^ Archer, a Danaher Company, Boulder, CO; ^6^ Department of Pediatrics and Cancer Center, School of Medicine, Texas Tech University Health Sciences Center, Lubbock, TX; ^7^ Department of Pediatrics, Hospital for Sick Children and University of Toronto, Toronto, Canada; ^8^ Perelman School of Medicine at the University of Pennsylvania, Philadelphia, PA; ^9^ Dana-Farber/Boston Children's Cancer and Blood Disorders Center, Harvard Medical School, Boston, MA; ^10^ Department of Oncology, St Jude Children's Research Hospital, Memphis, TN

## Abstract

**PURPOSE:**

Relapsed high-risk neuroblastomas (NBLs) are enriched for targetable mutations in *ALK* and RAS-MAPK pathways, yet the prognostic effect of these aberrations and relevance of subclonal mutations at diagnosis remain undefined. We describe the spectrum and clinical significance of clonal and subclonal pathogenic alterations in high-risk NBL.

**METHODS:**

We developed a focused high-risk NBL sequencing panel including *ALK*, *NRAS*, *KRAS*, *HRAS*, *BRAF*, *PTPN11*, *TP53*, and *ATRX* genes for ultra-deep sequencing and applied this assay to 242 pretherapy tumors from patients enrolled on the phase III trial Children's Oncology Group ANBL0532. We assessed the effect of clonal and subclonal mutations on event-free survival (EFS) and overall survival (OS).

**RESULTS:**

*ALK*-activating mutations occurred in 21.5% of tumors (n = 52, 30 clonal, 22 subclonal), and 3.3% (n = 8) showed *ALK* amplification. EFS and OS for patients with any *ALK*-aberrant tumor were inferior to patients with wild-type (WT) *ALK* tumors (5-year OS 37.7% *v* 66.3%; hazard ratio [HR], 1.992; *P* = .0007). EFS and OS for patients with tumors harboring activating *ALK* mutations ≥5% variant allele frequency (VAF) were inferior to *ALK* WT (5-year OS 37.7% *v* 66.3%; HR, 1.966; *P* = .0041). The 5-year EFS and OS for patients with *ALK*-amplified tumors were 25.0%. RAS pathway mutations occurred in 7.9% of tumors (n = 19; four clonal, 15 subclonal), with EFS and OS for those with VAF ≥5% inferior to RAS-WT patients (5-year OS 19.1% *v* 60.0%; HR, 3.021; *P* = .0168).

**CONCLUSION:**

Ultra-deep sequencing of high-risk NBLs demonstrates that oncogenic aberrations are more prevalent at diagnosis than previously recognized. *ALK* and RAS pathway aberrations confer inferior outcomes in patients treated with contemporary therapy, emphasizing the need for novel therapeutic approaches.

## INTRODUCTION

Pediatric solid tumors harbor few somatic mutations at diagnosis,^1^ limiting incorporation of targeted therapy that could potentially improve outcomes and decrease treatment-related morbidity. Neuroblastoma, a malignancy of the developing peripheral nervous system, has heterogeneous molecular features and outcomes, and patients with high-risk neuroblastoma (NBL) have approximately 50% survival despite dose-intensive multimodal therapy.^2^ HR-NBL is distinguished by features such as segmental chromosomal aberrations, *MYCN* amplification, and *ALK* alterations (mutation/amplification) in 14% of patients.^3-5^ In contrast, relapsed neuroblastomas demonstrate higher frequencies of *ALK* and RAS-MAPK pathway mutations,^6-8^ suggesting enrichment of selected subclonal mutations as a mechanism of therapy resistance. Serial studies of tissue and circulating tumor DNA (ctDNA) demonstrate evolution of these mutations during treatment,^9,10^ underscoring the need to apply deep next-generation sequencing (NGS) methodologies to understand whether they are present at diagnosis and evaluate their prognostic effect.

**CONTEXT**

**Key Objective**
Identify the prognostic effect and clinically relevant allele frequencies in pathogenic alterations of ALK and other oncogenic drivers at diagnosis in patients with high-risk neuroblastoma (NBL).
**Knowledge Generated**
Clonal and subclonal mutations (variant allele frequency ≥5%) in ALK confer inferior prognosis for patients treated with modern therapy, particularly when they co-occur with MYCN amplification. The presence of RAS pathway mutations at diagnosis also portends particularly poor outcomes.
**Relevance *(S.K. Bhatia)***
This study demonstrates the importance of risk-stratified management of patients with high-risk NBL based on the molecular alterations found at diagnosis, and, if validated, the need to identify innovative therapies for patients with *ALK*, RAS pathway, and *TP53* aberrations.*
*Relevance section written by *JCO* Associate Editor Smita K. Bhatia, MD, MPH, FASCO.

Aberrations in *ALK*, RAS-MAPK pathway genes, and *TP53* have been reported at diagnosis at relatively low frequencies and collectively are associated with inferior outcome, suggestive of a very-high-risk disease subtype.^11^ *ALK* is the most frequently mutated and tractable oncogene in high-risk NBL, where gene amplification or mutations in the tyrosine kinase domain (TKD; 85% of which occur at three hotspots at positions 1,174, 1,245, and, 1,275) cause constitutive protein activation and confer inferior outcomes.^4,12^ Our initial retrospective studies using Sanger sequencing identified clonal *ALK* mutations in 10% and amplifications in 4% of patients with high-risk NBL in historical Children's Oncology Group (COG) studies.^12^ With the incorporation of NGS and the ability to detect mutations at lower variant allele frequencies (VAFs), *ALK* aberrations were recently identified in 18.4% of patients (13.9% mutations, 4.5% amplifications).^13^ Previous studies defined clonal mutations as those with VAF ≥20% and subclonal as <20% and showed the prognostic effect of clonal *ALK* mutations only.^13^ The clinical relevance of subclonal mutations at diagnosis remains undefined, with conflicting reports from retrospective studies that used multiple methodologies with varying sensitivities for low-frequency variants.^13-16^ The prognostic implications of clonal versus subclonal RAS pathway and *TP53* mutations, and *ATRX* aberrations, have not been reported.

Our preclinical and early-phase trials^17-21^ provided proof-of-concept for integration of *ALK* sequencing at diagnosis and nonrandom assignment of patients harboring *ALK* amplification or activating mutations to treatment with the third-generation ALK inhibitor lorlatinib combined with standard therapy (arm E) in the COG phase III ANBL1531 trial (ClinicalTrials.gov identifier: NCT03126916). As sequencing methodologies improve and low-frequency variants are identified from tumor tissues, it is essential to define clinical relevance to inform treatment decisions. Here, we sought to ascertain the incidence and effect of aberrations in key neuroblastoma-associated genes from the completed COG phase III ANBL0532 trial (ClinicalTrials.gov identifier: NCT00567567)^22^ using a uniform, highly sensitive and specific ultra-deep sequencing approach. We report the landscape of these potentially targetable genetic aberrations at diagnosis and define VAF thresholds with prognostic significance.

## METHODS

### Patient Cohort

Patient eligibility criteria for ANBL0532 have been previously reported.^22^ Patient enrollment and sample preparation are detailed in the Data Supplement (Methods, online only).

### High-Risk NBL NGS Gene Panel Assay

We developed a custom ArcherDx (Integrated DNA Technologies) VariantPlex panel using anchored multiplex polymerase chain reaction to identify structural and sequence variation in the targeted regions and unique molecular identifiers to allow for ultra-deep sequencing with error correction. The panel was designed to target select regions from nine clinically relevant genes in neuroblastoma: *ALK*, *ATRX*, *TP53*, *NRAS*, *HRAS*, *KRAS*, *BRAF*, *PTPN11*, and *MYCN* (Data Supplement, Table S1). Genes were selected after comprehensive review of published high-risk NBL genomic data sets^5,6,8,23^ and internal patient cohorts at the Children's Hospital of Philadelphia.^9,10^ Selected genes were prioritized on the basis of observed mutational frequency at diagnosis and relapse, and potential for targeted treatment. Small panel size was optimized for ultra-deep sequencing of limited diagnostic tissue to determine whether genes enriched at relapse are present subclonally at diagnosis and whether their presence confers prognostic significance. We included a 1:4 dilution of *MYCN* primers to allow for accurate assessment of *MYCN* amplification without sacrificing read depth of other covered regions due to abundance of *MYCN* reads. Panel optimization and validation and sequencing metrics are provided in the Data Supplement (Methods).

### Variant Interpretation and Selection

Variant calling, annotation, and selection methods are described in the Data Supplement (Methods). Pathogenic variants were selected as follows: For mutations in *ALK*, we included hotspots (F1174, F1245, and R1275) and mutations reported to cause protein activation.^12^ We conducted molecular dynamic modeling to predict the effect of remaining mutations on protein activity and included only those predicted to cause ALK activation.^9,12,24^ For tumors with >one activating *ALK* mutation, the mutation with highest VAF was selected to characterize clonal status; the patient with both *ALK* amplification and a subclonal *ALK* mutation was characterized as *ALK*-amplified. For *TP53*, variants were queried in ClinVar and the ClinGen *TP53* Expert Panel Specifications to the American College of Medical Genetics and Genomics/Association for Molecular Pathology Variant Interpretation Guidelines for *TP53* V1.2.^25,26^ For RAS pathway mutations, we included only those mutations reported more than once in the COSMIC database. For *ATRX*, mutations were selected only if the alternative lengthening of telomeres (ALT) analysis of the tumor was also positive. Samples were considered to maintain telomeres via ALT if they were C-circle–positive, as previously described.^27,28^ In one sample with an *ATRX* deletion and three subclonal mutations that was C-circle–negative, we determined that the tumor was ALT-positive using two orthogonal methods, ALT-associated promyelocytic leukemia bodies and ultra-bright telomere foci using archived FFPE tissue sections, as previously described.^27,29^

### Copy Number Variation Calling

Details of copy number variation calling are provided in the Data Supplement (Methods). *MYCN* status was determined by centralized COG fluorescence in situ hybridization (FISH) testing.^30^ *MYCN* amplification calls were concordant between FISH and NGS for 96.2% of samples (230/239). In the nine discordant samples, six were consistent with a gain (<10 copies; Data Supplement, Fig S1), two showed copy number neutrality, and one showed high-level *MYCN* amplification (Data Supplement, Fig S2), in contrast to COG data. In cases of discrepancy, the COG data were used, as FISH is currently considered the gold standard for clinical annotation. For three patients lacking central *MYCN* FISH results, we determined *MYCN* status on the basis of sequencing results.

Details of statistical analysis are provided in the Data Supplement (Methods).

## RESULTS

### Patient Characteristics

Of the 652 eligible patients enrolled on ANBL0532, 250 diagnostic tumor tissues were available for this study. Limited material was obtained from initial diagnostic biopsies and required clinical biomarker testing centrally performed before ANBL0532 enrollment further depleted available material. DNA from 242 of 250 tumors was successfully sequenced and used for analysis. The sequenced study patient cohort was reflective of the full ANBL0532 trial cohort (Table 1). Within the study cohort, 42.1% (n = 102) were randomly assigned to tandem transplant, and 63.2% (n = 153) received postconsolidation dinutuximab-based immunotherapy. Tumors were *MYCN*-amplified in 50.4% (n = 122). The median follow-up time for the 107 patients without an event in the study cohort (N = 242 patients) was 9.6 years. There was a significant difference in the distribution of International Neuroblastoma Staging System (INSS) stage (*P* = .0017) with 21.7% of patients having INSS stage III (n = 13/60) and 76.7% (n = 46/60) with stage IV in patients with *ALK*-aberrant disease, versus 6.6% (n = 12/182) with stage III and 91.2% (n = 166/182) with stage IV disease in patients with *ALK*-wild-type (WT) tumors (Table 1).

**TABLE 1.** Patient Characteristics

Factor	Full ANBL0532 Cohort, No. (%)	Study Cohort, No. (%)	ALK Wild Type, No. (%)	All ALK Aberrations, No. (%)	*P*
Sample size of cohort	652	242	182	60	
Age					.6394
<547 days old at diagnosis	79 (12.1)	32 (13.2)	23 (12.6)	9 (15.0)	
≥547 days old at diagnosis	573 (87.9)	210 (86.8)	159 (87.4)	51 (85.0)	
INSS stage					.0017^a^
Stage I, II	7 (1.1)	4 (1.7)	4 (2.2)	0	
Stage III	68 (10.4)	25 (10.3)	12 (6.6)	13 (21.7)	
Stage IVS	3 (0.5)	1 (0.4)	0	1 (1.7)	
Stage IV	574 (88.0)	212 (87.6)	166 (91.2)	46 (76.7)	
Primary site (dx)					.1691^a^
Adrenal/abdominal	558 (85.6)	215 (88.8)	157 (86.3)	58 (96.7)	
Thorax	40 (6.1)	15 (6.2)	14 (7.7)	1 (1.7)	
Paraspinal, other	8 (1.2)	1 (0.4)	1 (0.6)	0	
Other	46 (7.1)	11 (4.6)	10 (5.5)	1 (1.7)	
Sex					.4589
Female	286 (43.9)	107 (44.2)	78 (42.9)	29 (48.3)	
Male	366 (56.1)	135 (55.8)	104 (57.1)	31 (51.7)	
Race					1.0000^a^
American Indian/Alaska Native	1 (0.2)	0	0	0	
Asian	23 (3.9)	7 (3.2)	5 (3.0)	2 (3.6)	
Native Hawaiian or other Pacific Islander	4 (0.7)	1 (0.5)	1 (0.6)	0	
Black or African American	82 (13.9)	30 (13.5)	23 (13.8)	7 (12.7)	
White	482 (81.4)	184 (82.9)	138 (82.6)	46 (83.6)	
Unknown or not reported	60	20	15	5	
Ethnicity					.3063
Hispanic or Latino	77 (12.3)	35 (15.1)	24 (13.7)	11 (19.3)	
Not Hispanic Latino	548 (87.7)	197 (84.9)	151 (86.3)	46 (80.7)	
Unknown	27	10	7	3	
MYCN^b^					<.0001
Amplified	249 (43.2)	122 (50.4)	78 (42.9)	44 (73.3)	
Not amplified	327 (56.8)	120 (49.6)	104 (57.1)	16 (26.7)	
Unknown	76	0	0	0	
End-induction response					.6027
CR/VGPR	277 (45.0)	112 (46.5)	81 (44.5)	31 (52.5)	
PR	213 (34.6)	85 (35.3)	68 (37.4)	17 (28.8)	
NR/MR	79 (12.8)	15 (6.2)	12 (6.6)	3 (5.1)	
PD	46 (7.5)	29 (12.0)	21 (11.5)	8 (13.6)	
Unknown	37	1	0	1	

Abbreviations: CR, complete response; FISH, fluorescence in situ hybridization; INSS, International Neuroblastoma Staging System; MR, minor response; NR, no response; PD, progressive disease; PR, partial response; VGPR, very good partial response.

^a^ Denotes use of Fisher's exact test.

^b^ Three patients lacking central *MYCN* FISH results were categorized using sequencing results (two not amplified, one amplified).

### Frequency of Pathogenic Alterations at Diagnosis

**FIG 1.**
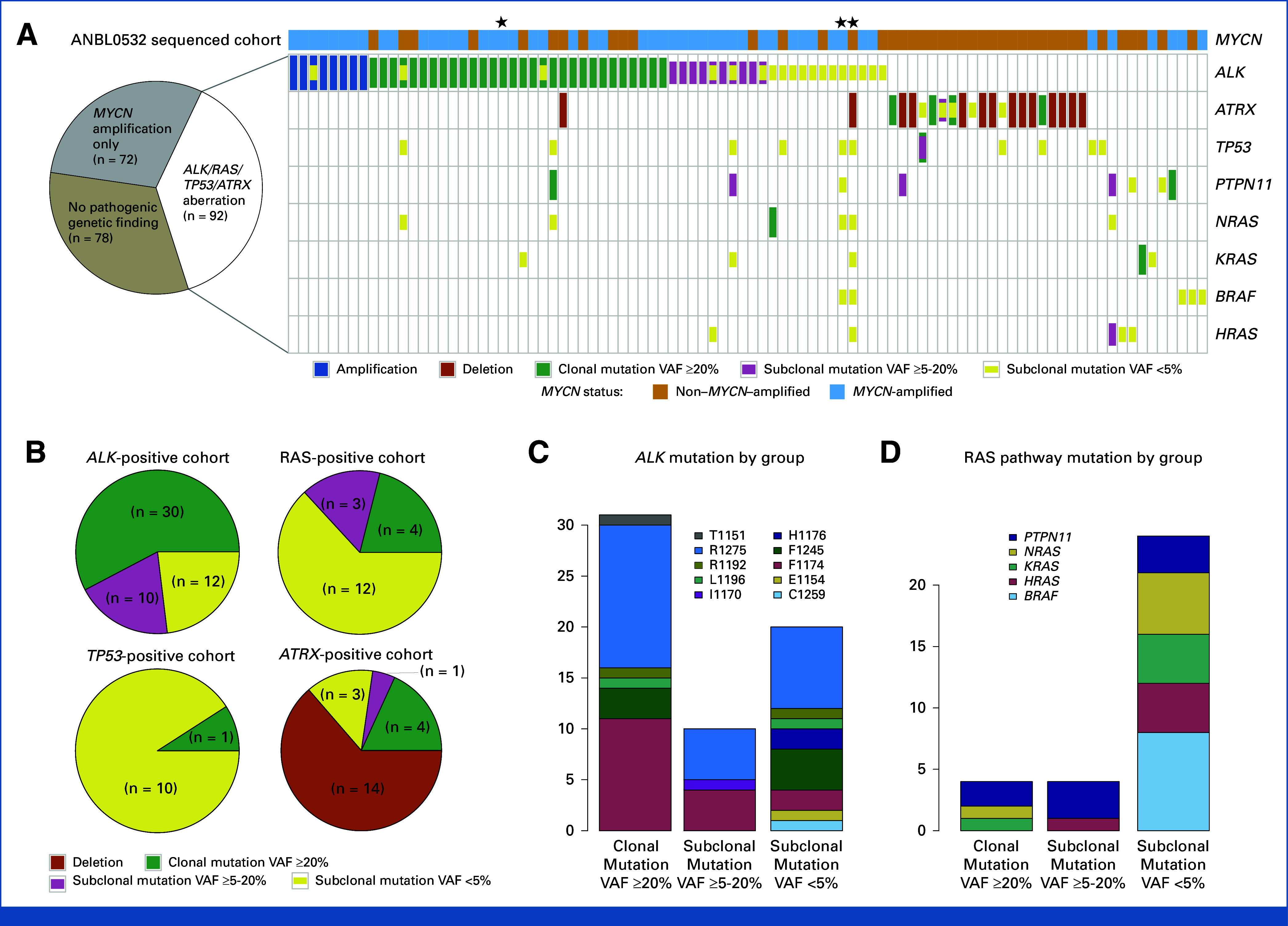
Pathogenic alterations in ANBL0532 cohort. (A) Oncoplot of pathogenic alterations in the cohort. Each column demonstrates a patient, with each row corresponding to a gene, as annotated. Star indicates patients with tumors harboring >one concurrent *ALK* mutation of the type depicted. Patients with no alterations identified are not depicted. (B) Patient tumors classified as harboring clonal (VAF ≥20%) and subclonal (VAF ≥5%-20%, VAF <5%) mutations. Panels (C and D) depict the full range of observed mutations, some of which co-occurred in patients. (C) *ALK* mutations per VAF group; (D) RAS pathway genes mutated per VAF group. VAF, variant allele frequency.

Using our custom NGS panel, pathogenic alterations were identified in tumors from 38% (n = 92/242) of patients (Fig 1A; Data Supplement, Table S2). We detected *ALK* aberrations in 24.8% (n = 60) of tumors. Activating *ALK* mutations were present in 21.5% (n = 52) of tumors, with 12.4% (n = 30) harboring an *ALK* mutation with VAF ≥20%, 4.1% (n = 10) harboring mutations at VAF ≥5%-20%, and 5.0% (n = 12) with mutations at VAF <5% (Fig 1B). In tumors with *ALK* mutations ≥5% VAF, 7.5% (n = 3/40) harbored non-hotspot activating mutations versus 25% (n = 3/12) with non-hotspot activating mutations in tumors with subclonal *ALK* mutations with a VAF <5%. All included novel ALK TKD mutations (Fig 1C) were predicted to be activating using in silico computational algorithms.^9,12,24^ *ALK* amplification was detected in 3.3% of tumors (n = 8); all *ALK*-amplified tumors had concurrent *MYCN* amplification. One patient had an *ALK*-amplified tumor with concurrent subclonal *ALK* R1192W oncogenic mutation (VAF 0.1%). Two or more activating *ALK* mutations were detected in nine tumors. One patient's tumor harbored two clonal mutations (F1174L and R1192G) at high VAF (31.6% and 64.8% VAF, respectively). In two patient tumors, a clonal *ALK* mutation co-occurred with a low VAF (<5%) mutation, and in three tumors, a subclonal *ALK* mutation (VAF ≥5%-20%) co-occurred with a low VAF (<5%) mutation. Two tumors harbored multiple low VAF (<5%) *ALK* mutations (Fig 1A). The *ALK*-aberrant stage III cohort consisted of two (15.4%) tumors with *ALK* amplification, seven (53.8%) with a clonal *ALK* mutation at VAF ≥20%, two with *ALK* mutations ≥5%-20% VAF, and two with mutations <5% VAF.

We identified mutations across all represented RAS pathway genes (*NRAS*, *KRAS*, *HRAS*, *PTPN11*, *BRAF*; Fig 1D), with pathogenic variants in 7.9% of tumors (n = 19), including 1.7% (n = 4) present at VAF ≥20% and 6.2% (n = 15) at <20% VAF (Figs 1A and 1B). Pathogenic variants in *TP53* were identified in 4.5% (n = 11) of tumors (Fig 1B). Of the 11, only one tumor harbored a clonal *TP53* mutation (VAF = 39.8%), with a second concurrent mutation with VAF 7.41%. The remaining 10 tumors harbored low-frequency mutations (VAF <5%), including two with multiple concurrent low subclonal (VAF <5%) mutations (Fig 1A). We identified loss-of-function aberrations of *ATRX* in 9% of tumors (n = 22), all of which were positive for ALT-pathway activation and none showing *MYCN* amplification. These occurred as N-terminal deletions in 5.7% of the cohort (n = 14) and mutations in 3.3% (n = 8; Figs 1A and 1B).

### Aberrations in *ALK* and the RAS-MAPK Pathway (VAF of ≥5%) Confer Inferior Prognosis

**FIG 2.**
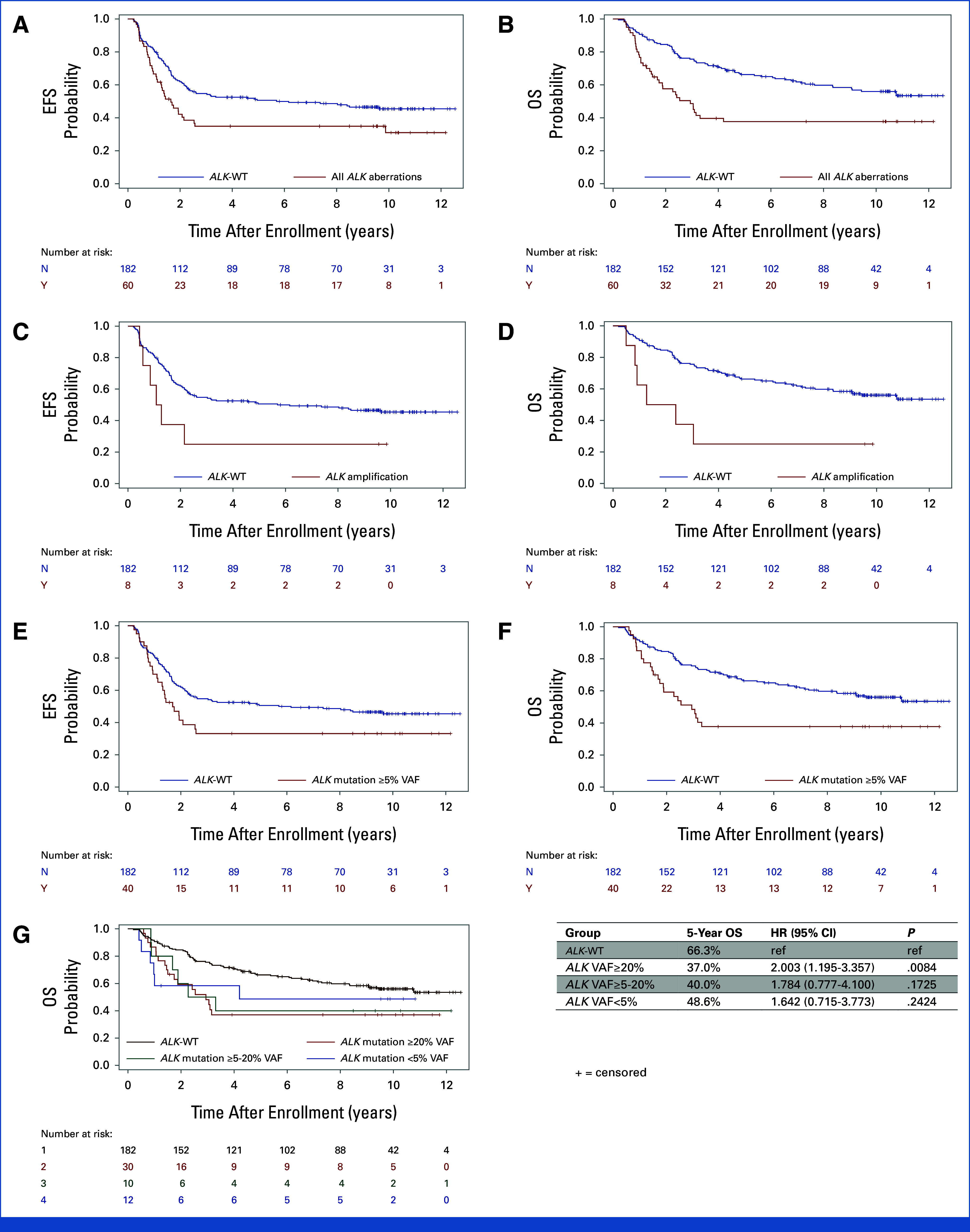
EFS and OS with *ALK* aberrations. (A) EFS of patients with tumors harboring all *ALK* aberrations compared with patients with *ALK*-WT tumors. (B) OS of patients with tumor harboring all *ALK* aberrations compared with patients with *ALK*-WT tumors. (C) EFS of patients with tumors harboring *ALK* amplification compared with patients with *ALK*-WT tumors. (D) OS of patients with tumors harboring *ALK* amplification compared with patients with *ALK*-WT tumors. (E) EFS of patients with tumors harboring *ALK* mutations with VAF ≥5% compared with patients with *ALK*-WT tumors. (F) OS of patients with tumors harboring *ALK* mutations with VAF ≥5% compared with patients with *ALK*-WT tumors. (G) OS of patients with tumors harboring *ALK* mutations stratified by VAF compared with patients with *ALK*-WT tumors. Table shows HR, 95% CI, and *P* values for each VAF subgroup compared with the WT. EFS, event-free survival; HR, hazard ratio; OS, overall survival; ref, reference; VAF, variant allele frequency; WT, wild type.

The presence of any *ALK* aberration portended inferior outcome, with a 5-year event-free survival (EFS) of 34.9% versus 50.6% in patients with *ALK*-WT tumors (Fig 2A; hazard ratio [HR], 1.556 [95% CI, 1.071 to 2.261]; *P* = .0203). The 5-year overall survival (OS) for patients with tumors harboring any activating *ALK* aberration was 37.7% versus 66.3% in *ALK*-WT tumors (Fig 2B; HR, 1.992 [95% CI, 1.337 to 2.967]; *P* = .0007). Patients with tumors harboring *ALK* amplification had particularly poor outcomes, with a 5-year EFS of 25.0% versus 50.6% in *ALK*-WT (Fig 2C; HR, 2.021 [95% CI, 0.884 to 4.619]; *P* = .0953) and a 5-year OS of 25.0% versus 66.3% for *ALK*-WT (Fig 2D; HR, 3.018 [95% CI, 1.310 to 6.951]; *P* = .0095).

To inform Arm E of the COG phase III trial where a VAF ≥5% is used to assign patients to lorlatinib, we evaluated the effect of low-frequency mutations on outcome. In total, 16.5% (n = 40) of patient tumors harbored activating *ALK* mutations with VAF ≥5%. These patients had a 5-year EFS of 33.2% versus 50.6% in the *ALK*-WT group (Fig 2E; HR, 1.539 [95% CI, 0.997 to 2.377]; *P* = .0518) and a 5-year OS of 37.7% versus 66.3% in the *ALK*-WT group (Fig 2F; HR, 1.966 [95% CI, 1.240 to 3.118]; *P* = .0041). We stratified patients by mutant *ALK* VAF, differentiating outcomes between cohorts with mutations at VAF >20%, ≥5%-20%, and <5%. Although there was no significant difference in EFS (Data Supplement, Fig S3), OS was significantly different (Fig 2G). Patients with mutant *ALK* VAF ≥20% in their tumor had a 5-year OS of 37.0% versus a 5-year OS of 66.3% in the *ALK*-WT cohort, and the cohort with VAF 5%-20% demonstrated a similar trend, with a 5-year OS of 40.0%, although a statistically significant difference was not detected in this small patient sample. Patients with tumors harboring *ALK* mutations at VAF <5% had outcomes like patients with *ALK*-WT tumors. Compared with patients with *ALK*-WT tumors, patients with *ALK*-aberrant tumors (VAF ≥5% or amplifications) fared worse among those treated with either single or tandem transplant (Data Supplement, Fig S4). We observed differential effect on prognosis of specific *ALK* hotspot variants (VAF ≥5%). Patients with an F1245 mutation (n = 3) had the worst outcome (5-year EFS and OS 0%), those with F1174 mutations (n = 15) had a 5-year EFS of 13.3% and an OS of 20.0%, and those with R1275 mutations (n = 19) had a 5-year EFS of 49.1% and an OS of 54.7% versus a 5-year EFS of 50.6% and an OS of 66.3% in patients with *ALK*-WT tumors (Data Supplement, Fig S5). The F1174 mutation was significantly enriched in *MYCN*-amplified tumors (Data Supplement, Table S3). Patients with *ALK*-aberrant stage III disease (n = 13) had a 5-year EFS of 60.6% versus 83.3% in patients with stage III *ALK*-WT disease (n = 12; HR, 2.750 [95% CI, 0.532 to 14.216]; *P* = .2274). The 5-year OS for patients with *ALK*-aberrant stage III disease was 59.8% versus 91.7% for stage III patients with *ALK*-WT disease (HR, 5.572 [95% CI, 0.649 to 47.808]; *P* = .1173).

**FIG 3.**
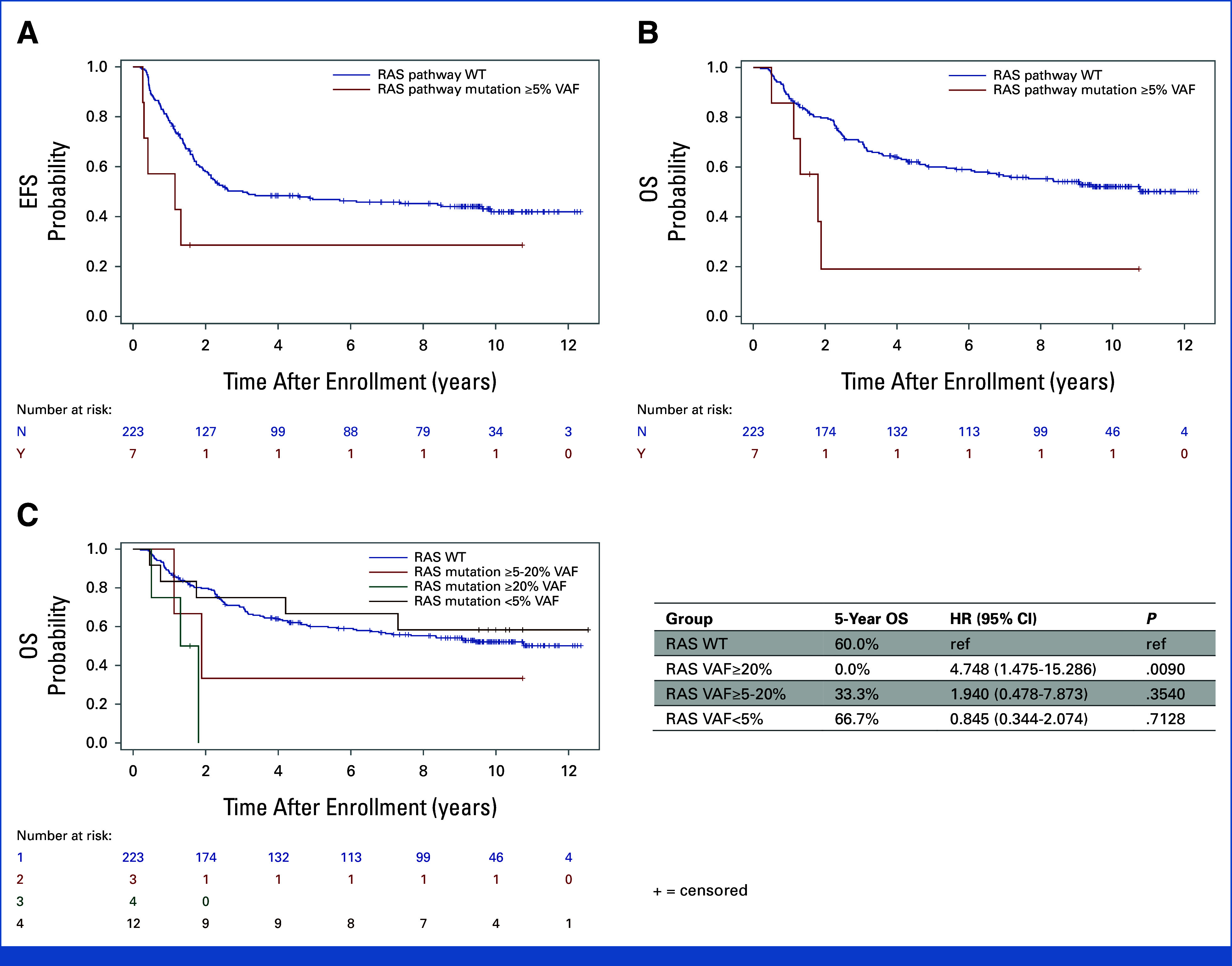
EFS and OS with RAS pathway mutations. (A) EFS of patients with tumors harboring RAS pathway mutations with VAF ≥5% compared with patients with RAS pathway WT tumors. (B) OS of patients with tumors harboring RAS pathway mutations with VAF ≥5% compared with patients with RAS pathway WT tumors. (C) OS of patients with tumors harboring RAS pathway mutations stratified by VAF compared with patients with RAS pathway WT tumors. Table shows HR, 95% CI, and *P* values for each VAF subgroup compared with the WT. EFS, event-free survival; HR, hazard ratio; OS, overall survival; ref, reference; VAF, variant allele frequency; WT, wild type.

Although there was no difference in outcome when comparing the entire RAS pathway mutant versus RAS pathway WT group (Data Supplement, Figs S6A and S6B), there were significant differences in outcomes for patients with tumors harboring an RAS pathway activating mutation at VAF ≥5%, with a 5-year EFS of 28.6% versus 46.8% in the RAS-WT group (Fig 3A; HR, 2.508 [95% CI, 1.023 to 6.150]; *P* = .0444). The 5-year OS for this cohort was 19.1% versus 60.0% for RAS-WT (Fig 3B; HR, 3.021 [95% CI, 1.221 to 7.474]; *P* = .0168). Further stratification on the basis of VAF resulted in subgroups that were too small for robust statistical analysis; however, patients with tumors harboring RAS pathway mutations ≥20% VAF had a 5-year OS of 0.0% (n = 4) versus 60.0% in RAS-WT (n = 223), and patients with RAS mutations ≥5%-20% VAF (n = 3) had a 5-year OS inferior to WT patients (Fig 3C; Data Supplement, Fig S6C).

### Co-Occurrence of *MYCN* Amplification and *ALK*-Aberrations Is Frequent and Portends Inferior Outcome

**FIG 4.**
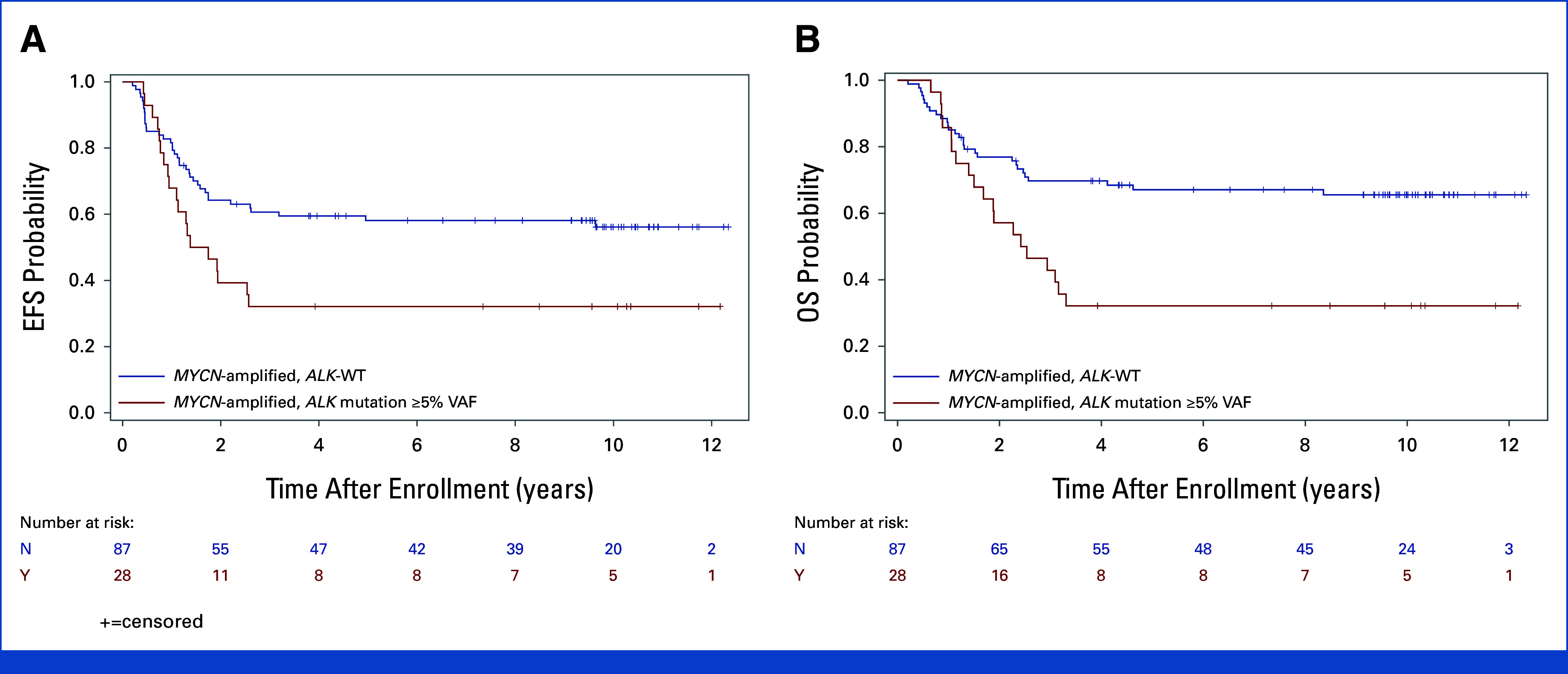
EFS and OS of patients with concurrent *MYCN* amplification and *ALK* mutation. (A) EFS of patients with tumors harboring *MYCN* amplification and *ALK* mutations with VAF ≥5% compared with patients with *MYCN*-amplified and *ALK*-WT tumors. (B) OS of patients with tumors harboring *MYCN* amplification and *ALK* mutations with VAF ≥5% compared with patients with *MYCN*-amplified and *ALK*-WT tumors. EFS, event-free survival; OS, overall survival; VAF, variant allele frequency; WT, wild type.

Consistent with findings in the SIOPEN cohort,^13^ *MYCN* amplification was more frequent in the *ALK*-aberrant cohort, occurring in 73.3% of patients with tumors harboring an *ALK* aberration versus 42.9% in the *ALK*-WT group (*P* < .0001; Table 1). *MYCN* amplification was more frequent in the *ALK* mutation–only cohort, occurring in 69.2% (n = 36) versus 42.9% (n = 78) in *ALK*-WT patients (*P* = .0008). The combination of *MYCN* amplification and *ALK* mutation (VAF ≥5%) was particularly deleterious, resulting in a 5-year EFS of 32.1% versus 58.1% in patients with *MYCN*-amplified, *ALK*-WT tumors (HR, 1.897 [95% CI, 1.088 to 3.306]; *P* = .0240) and a 5-year OS of 32.1% versus 67.1% in patients with *MYCN*-amplified, *ALK*-WT disease (HR, 2.367 [95% CI, 1.323 to 4.236]; *P* = .0037; Fig 4). *ALK* mutations (VAF ≥5%) that occurred in tumors without *MYCN* amplification did not affect prognosis (Data Supplement, Fig S7), although the small patient numbers (n = 12) preclude definitive conclusions.

### Prognostic Effect of *TP53* and *ATRX* Aberrations

**FIG 5.**
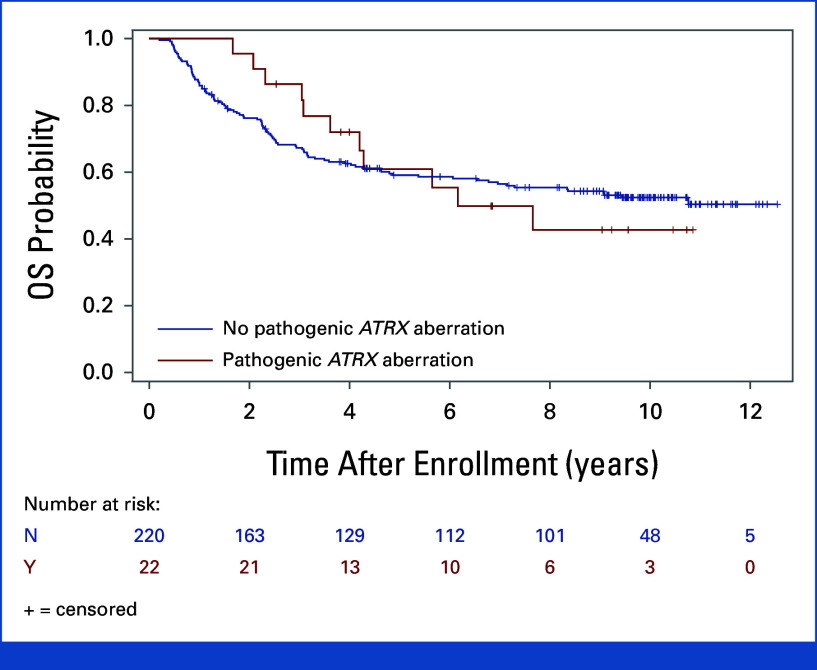
OS of patients with *ATRX* aberrations. OS of patients with tumors harboring pathogenic *ATRX* aberrations compared with patients with *ATRX*-WT tumors. OS, overall survival.

Evaluation of *TP53* mutations on outcome was limited due to small numbers. The single patient with a tumor harboring a pathogenic clonal *TP53* variant died of relapsed disease <2 years after diagnosis. The presence of *ATRX* aberration did not appear to affect outcome, as patients with detectable *ATRX* aberrations had a 5-year EFS of 49.6% versus 46.5% in the *ATRX*-WT group (Data Supplement, Fig S8; HR, 0.844 [95% CI, 0.466 to 1.527]; *P* = .5747) and a 5-year OS of 60.9% versus 59.1% in *ATRX*-WT (Fig 5; HR, 0.403 [95% CI, 0.138 to 1.180]; *P* = .0972, adjustment for nonproportional hazards required).

We investigated whether cohorts stratified by molecular features show differential response to induction treatment. There was no significant difference in response to induction chemotherapy (per the 1993 International Neuroblastoma Response Criteria^31^ used in the ANBL0532 study) on the basis of *ALK*, RAS pathway, or *ATRX* aberration status (Data Supplement, Table S4). A synopsis of findings by patient subgroup is provided in the Data Supplement (Table S5).

## DISCUSSION

We leveraged a modern patient cohort with high-risk NBL and the longest reported outcome data^22^ to establish the incidence of potentially tractable genetic aberrations at diagnosis. We identified pathogenic *ALK* alterations in 24.8% of tumors (21.5% mutations and 3.3% amplification). The overall incidence is higher than previous reports,^13,14^ which may stem from our use of NGS methodology and a purposely restricted gene panel to maximize sequencing depth. Additionally, we included non-hotspot *ALK* TKD mutations predicted to be activating, consistent with the approach used on COG ANBL1531 for assignment to lorlatinib. Our data show the differential effect of the three *ALK* hotspot mutations, with patients harboring somatic F1245 or F1174 mutations faring worse than those with R1275 mutations or *ALK*-WT disease. This is consistent with our biochemical studies showing the strongest effect of F1174 and F1245 mutations on ALK catalytic activity.^12^

Our data show a higher frequency of co-occurrence of *MYCN* amplification and *ALK* mutations, with particularly poor outcomes for these patients. The NANT study of lorlatinib demonstrated a lack of sustained response to lorlatinib monotherapy in young patients with *ALK*-aberrant *MYCN*-amplified disease, although most were not treated at the recommended phase II dose.^32^ This is not entirely surprising since *MYCN* amplification at relapse remains the most influential biomarker of dismal outcome due to rapid disease progression.^33^ The role of *ALK* aberrations in the absence of concurrent *MYCN* amplification requires further study, as our limited data are consistent with some observations in heterogeneous cohorts that included non–high-risk patients.^15,16^ These data support the importance of testing ALK inhibition in a therapy-naïve population with dismal outcomes and the need to develop novel therapeutic approaches.

Our results support the utilization of VAF ≥5% for assignment of patients with *ALK*-aberrant disease to therapy with lorlatinib. Outcomes for patients with tumors harboring either *ALK* or RAS pathway subclonal mutations between 5% and 20% appear inferior to those of patients with WT tumors or mutations <5% VAF. These findings may not have been statistically significant due to the relatively small sample size, and further studies are necessary to definitively establish the prognostic value of these subclonal mutations. Phase III testing of lorlatinib in frontline therapy and implementation of ultra-deep sequencing of diagnostic tumors will provide additional data on evolution and elimination of subclones and effect on survival, informing future studies. Importantly, studies of serial ctDNA, which more comprehensively reflect tumor genetic heterogeneity, are essential to further interrogate and track the prognostic significance of evolving subclones at diagnosis and during therapy.^9^

Our data on *ALK* aberrations are in contrast to both the SIOPEN and GPOH cohorts, which demonstrated an effect of only clonal *ALK* mutations on OS^13,14^; however, these studies did not further stratify VAF <20% as we did in this study. In addition, limited panel sequencing does not allow for correction of cancer cell content, and percutaneous needle core biopsy, which is now widely used at diagnosis, provides restricted sampling of heterogeneous tumors. It is likely that subclonal mutations exist within tumors harboring both higher and lower oncogenic VAFs. Furthermore, the 5-year OS for patients with *ALK*-WT tumors in both European cohorts was approximately 50%, whereas those treated on the ANBL0532 COG trial had a 5-year OS >60%.^13,14^

To our knowledge, this is the first study to report a significant enrichment of pathogenic *ALK* aberrations in patients with localized stage III disease. Although small sample size precludes definitive assessment of prognosis, 38.5% of these patients died versus 8.3% in the *ALK*-WT group. Major cooperative group trials differ in risk classification and treatment approach for patients with locoregional disease; additionally, patient outcomes vary depending on underlying tumor biology and genomics.^34-36^ Our data suggest that *ALK* aberrations in patients with nonmetastatic disease may be an adverse prognostic factor and treatment with an ALK inhibitor should be considered in future studies.

RAS pathway mutations were identified in 7.9% of patients, with 2.9% harboring mutations with a VAF ≥5%, consistent with a previous study that used a cutoff of 5% VAF.^11^ Likewise, the incidence of *ATRX* aberrations is consistent with previous reports.^3,37^ Only one tumor harbored a clonal *TP53* mutation, a frequency lower than previous reports,^11^ which may be due to our strict filtering criteria. *MYCN* amplification and *ATRX* aberrations were mutually exclusive, as previously reported.^38^ The OS curves for patients with *ATRX* aberrations crossed those for patients with *ATRX*-WT tumors, potentially reflective of the more indolent nature of *ATRX*-aberrant disease that is associated with better clinical course earlier on, but ultimately poor outcome.^37,38^ We observed numerous cases with co-occurrence of subclonal mutations across different gene pathways, yet there were only two cases of co-occurrence of clonal mutations across gene groups: one patient with clonal *ALK* and *PTPN11* mutations and one with clonal *ALK* mutation and *ATRX* deletion.

Our findings support early molecular stratification of patients with high-risk NBL with tumors found to have *ALK*, RAS pathway, or *TP53* aberrations to an ultra-high-risk group.^11^ These patients have inferior outcomes despite the current dose-intensive multimodal therapy that causes significant short- and long-term toxicities. Innovative therapies and optimized integration of frontline targeted therapies are essential for these patients.

## Data Availability

A data sharing statement provided by the authors is available with this article at DOI https://doi.org/10.1200/JCO-24-02407.
